# Diagnostic and prognostic utility of an inexpensive rapid on site malaria diagnostic test (ParaHIT *f*) among ethnic tribal population in areas of high, low and no transmission in central India

**DOI:** 10.1186/1471-2334-5-50

**Published:** 2005-06-21

**Authors:** Neeru Singh, AK Mishra, MM Shukla, SK Chand, Praveen Kumar Bharti

**Affiliations:** 1 Malaria Research Centre (ICMR) RMRCT Campus, Nagpur Road Jabalpur (M.P.), 482003 India

## Abstract

**Background:**

Malaria presents a diagnostic challenge in most tropical countries. Rapid detection of the malaria parasite and early treatment of infection still remain the most important goals of disease management. Therefore, performance characteristics of the new indigenous ParaHIT *f *test (Span diagnostic Ltd, Surat, India) was determined among ethnic tribal population in four districts of different transmission potential in central India to assess whether this rapid diagnostic test (RDT) could be widely applied as a diagnostic tool to control malaria. Beyond diagnosis, the logical utilization of RDTs is to monitor treatment outcome.

**Methods:**

A finger prick blood sample was collected from each clinically suspected case of malaria to prepare blood smear and for testing with the RDT after taking informed consent. The blood smears were read by an experienced technician blinded to the RDT results and clinical status of the subjects. The figures for specificity, sensitivity, accuracy and predictive values were calculated using microscopy as gold standard.

**Results:**

The prevalence of malaria infection estimated by RDT in parallel with microscopy provide evidence of the type of high, low or no transmission in the study area. Analysis revealed (pooled data of all four epidemiological settings) that overall sensitivity, specificity and accuracy of the RDT were >90% in areas of different endemicity. While, RDT is useful to confirm the diagnosis of new symptomatic cases of suspected *P. falciparum *infection, the persistence of parasite antigen leading to false positives even after clearance of asexual parasitaemia has limited its utility as a prognostic tool.

**Conclusion:**

The study showed that the ParaHIT *f *test was easy to use, reliable and cheap. Thus this RDT is an appropriate test for the use in the field by paramedical staff when laboratory facilities are not available and thus likely to contribute greatly to an effective control of malaria in resource poor countries.

## Background

Malaria presents a diagnostic challenge in most tropical countries [1] and diagnosis of malaria still relies predominantly upon clinical presentation and the century old technique of microscopic examination of blood smears. Diagnosis by clinical symptoms alone is highly unreliable [2]. Microscopy is labour intensive, requires significant skills and time, which caused therapeutic delays. These diagnostic limitations affects the medical care provided, as Malaria is a potentially fatal disease, usually curable if diagnosed quickly [3,4]. The urgency and importance of obtaining results quickly from the examination of blood samples from patients with suspected acute malaria is now made possible with the introduction of rapid malaria diagnostic tests (RDTS) [2]. Very few studies have evaluated a rapid test in different epidemiological settings. This is important since a test may be valid but expensive or valid and reliable in laboratory/ hospital clinic but with several inconveniences to use in the field or very easy but with an unacceptably low validity [5]. A new indigenous test, the cheapest available so far, ParaHIT *f *has recently been introduced in the national programme for malaria control in remote and inaccessible villages of Central India. We conducted a study to assess if this RDT is reliable, simple and practical in varied epidemiological situation in hospital and field. Our objective was to determine whether this RDT could be widely applied as a diagnostic tool to effective management and control of malaria.

## Methods

The ParaHIT *f *dipstick test (Code No. 25977, Span diagnostic Ltd, Surat, India) was evaluated as a diagnostic test in 4 districts of Central India (Fig. 1) with different transmission potential namely Jabalpur (Medical College hospital, a tertiary facility), Dindori (high transmission area), Mandla (very low/ no transmission due to intensive malaria control measures) and Korea district in Chhattisgarh (meso-endemic). The performance of the ParaHIT *f *for detecting *P. falciparum *infections was evaluated against microscopical examination of thick blood smears in field / hospital during April-August 2004.

### Study sites

#### Baigachak (Dindori district)

Dindori is highly malarious district contributing highest number of malaria cases (12%) in the state (6). The baiga tribe, living in an extremely difficult hilly terrain, with numerous hillocks (MSL. 980m). The population of 10 study villages ranged from 50–800. The villages are surrounded by reserved forests of Sal (*Shorea robusta*). The inhabitants are barely clothed and live in extremely primitive conditions. Medical facilities are non-existent. *Anopheles culicifacies *and *An. fluviatilis*, are two efficient vectors in the area. Seasonal transmission of *P. vivax *and *P. falciparum *are common. Chloroquine resistance against *P. falciparum *is prevalent. The villages are under indoor residual spray with synthetic pyrethroid (deltamethrin @20 mg/m^2^). All patients with fever or history of fever were screened for malaria in a mobile field clinic on a first come first serve basis.

#### Korea district (Chhattisgarh state)

District Korea is situated on the border of Madhya Pradesh in Chhattisgarh state. It also has dense forest and the terrain is undulating (MSL750m). The field clinic traveled 10 villages for screening malaria patients (gond ethnic tribe). The area is under 2 rounds of DDT spray (1 gm/m^2^). Other characteristics (vectors and transmission pattern) are the same as in Dindori.

#### Mocha (Mandla district)

Surveillance of fever cases was carried out in 10 villages (MSL – 475m). Transmission is seasonal and mainly due to *P. falciparum*. Other characteristics are the same as in Dindori.

#### Medical College hospital (Jabalpur district)

The subjects of the hospital based study were the suspected cases of malaria who presented with fever or history of fever, at the malaria clinic run by the Malaria Research Centre (MRC) at Medicine department of Government Medical College hospital. This hospital is the largest medical facility in Jabalpur district (MSL 394m) and serves both as a general hospital for the local people and as a referral hospital for the adjoining districts.

#### Sampling

A finger-prick blood sample was collected from each case, after verbal informed consent was obtained. This sample was used to prepare thick and thin smears and for testing with the ParaHIT *f *test. There were four teams of MRC, Field Station Jabalpur, each consisting of two field workers and one Research Scientist. A one hour workshop, including training in blood collection from finger prick, performance and interpretation of rapid test was conducted at MRC laboratory. All the team members had attended the workshop in the use of the RDTs. All the samples were tested in field/hospital unsupervised. The thick blood smears were also examined in the field. The smears were checked by a microscopist blinded to the clinical status of the subjects and to the results of the diagnostic tests. Parasitaemia was determined from the thick films by counting the number of parasites against 200 leucocytes and assuming that each subject had 8000 leucocytes/μl.

The ParaHIT *f *test was performed as per manufacturer's instructions [5]. The standard reading time is between 15–30 minutes. All adult subjects with *P. falciparum *were administered the standard oral dose of chloroquine (1500 mg chloroquine in 3 days) followed by primaquine (45 mg as a single dose). Children were given proportionally lower doses. Infants and pregnant women were not given primaquine.

To check that HRP-2 antigenaemia was cleared by treatment, finger prick blood samples were collected on day 10 post-treatment and tested using the RDT in parallel with blood smears. To minimize variability, one microscopist examined all the smears and one technician interpreted all the results of the rapid tests. A quality control procedure was put in place. All discordant results (between the RDT and the slide), all slides where only *P. falciparum *gametocytes were detected and random sample of 20% of the remaining slides were checked blind by an independent trained laboratory technician at MRC laboratory.

#### Ethical clearance and data analysis

The study protocol was approved by the ethics committee of the Malaria Research Centre (ICMR) Delhi.

The data were recorded and analyzed using statistical software (SPSS version 10.0, SPSS inc. Chicago 12). Once all the samples had been tested, specificity, sensitivity, predictive values and accuracy to the ParaHIT *f *were estimated using microscopy as gold standard [7,8]. Briefly, sensitivity was calculated as TP/ (TP+FN), specificity as TN/ (TN+FP), positive predictive value (PPV) as TP/ (TP+FP), negative predictive value (NPV) as TN/ (TN+FN) and accuracy as (TP+TN)/ number of all tests. The J-index i.e., the over all measure of reliability of a diagnostic test calculated as (TPxTN)-(FPxFN)/ (TP+FN) (TN+FP). The mixed infection of *P. vivax *and *P. falciparum *was treated as *P. falciparum *for the purpose of analysis.

## Results

The rates of fever and parasitaemia varied by study sites. The performance indices of RDT at each site is shown in Table 1.

### Baigachak, Dindori district (high transmission area)

Two hundred five of 467 patient's (43.9%) had positive thick films, with *P. falciparum *comprising 191, (40.9%) and *P. vivax *14 (3.0%). The asexual parasite density ranged from 640–280,000 parasites/μl (GMPD 9840 ± 7.38parasites/μl). The RDT detected malaria infections in 171 (89.5%) of 191 patients. The sensitivity of the rapid test was 90.2% (95%CI, 85–94) and specificity 83% (95%CI, 78–87%) compared to microscopy. The PPV and NPV were respectively 77% (95%CI, 71–83) and 93% (95%CI, 89–96).

In all 18 patients were positive by microscopy but negative by RDT and the parasitaemia in these patients ranged from 100–36,920 parasites/μl (GMPD 3715.35 ± 5.37). Only 94 cases were followed up on 10^th ^day, out of which 39 (41.5%) test were still positive post treatment. In 18 of the subjects (19%), the blood smears were also positive for *P. falciparum*. However, the RDT was positive only in 16 matching cases. 23 subjects were found false positive and 2 false negative with RDT. The sensitivity, specificity, PPV and NPV were respectively 84% (95% CI, 60.4–96.6), 69% (95% CI, 57.6–79.5), 41% (95% CI, 25.6–57.9) and 94.5% (95% CI, 84.9–98.9). The accuracy of test was 72.3% and J index 0.53 as compared to microscopy.

### Korea district (high to low transmission area, mero-endemic)

Of the 440 individuals screened, microscopic examination of the thick film detected 64 *P. falciparum*, 26 *P. vivax *and 1 mixed infection. The GMPD for *P. falciparum *was 323.59 ± 2.51 parasites/μl (range 80–7840 parasites/μl). The RDT detected malaria parasite in 57 subjects. The sensitivity and specificity of the test were 97% (95%CI, 88.5–99.6) and 95% (95% CI, 92–97) respectively with PPV 75% (95% CI, 64–85) and an NPV of 99.4% (95%CI, 98–100). When compared with microscopy, the accuracy of the test was 95.2% and J-index 0.92.

### Medical College hospital, Jabalpur (low transmission area)

Of the 208 patients screened, only 19 were infected with malaria. 7 *P. vivax *and 12 *P. falciparum*, of which 7 were cerebral malaria (CM) patients, comatose with various complications. All seven *P. falciparum *(CM) were also positive by RDT. However, results of the RDT were not used to guide treatment and antimalarial therapy was prescribed by the physician on the basis of clinical severity. The asexual parasitaemia ranged from 5000–100000 parasites/μl. The sensitivity of the test was 70% (95% CI, 35–93) with 93% specificity (95% CI, 88.4–96). The PPV and NPV were 33% (95%CI, 15–57) and 98.4% (95%CI, 95.4–99.7) respectively. The accuracy of the test was 92% and J-index 0.63 in comparison with gold standard.

### Mocha, Mandla district (no transmission area)

Only one hundred and two blood samples were screened by both the methods. Out of which only one was found RDT positive that too was smear negative.

Overall from 1217 subjects, 83 (6.8%) false positive were recorded. A prolonged re-examination of the slides from these 83 subjects, who were positive by the test, but apparently had negative slides, did not reveal the presence of *P. falciparum *trophozoites. 60 out of 83 subjects claimed to have taken antimalarials drugs in the preceding days. *P. falciparum *gametocytes only were seen in 14 cases of which 9 were RDT positive. Out of 47 subjects with *P. vivax *infections as detected by microscopy, only 5 were found positive for *P. falciparum *by the RDT.

## Discussion

Rapid, accurate diagnosis is necessary to effective management and control of malaria [2,9,10]. The strength of the present study is that it was performed in different epidemiological settings i.e. in one of the most difficult field conditions of baigachak and within the routine of tertiary Medical College hospital receiving persons of all ages with varying clinical severity and allowing the evaluation of the performance of the RDTs in all persons suspected to have malaria what ever their history i.e. recent malaria attack or anti-malaria drug intake in the previous days and clinical status (fever or not, severe or mild malaria). Therefore, the results obtained reflect the performance of the tests in a real situation.

From a malaria transmission perspective in baigachak, the RDT can play a key role in rapid diagnosis and prompt treatment of malaria in high transmission areas (where resistance to chloroquine also necessitates the use of more expensive alternate therapy). As RDT can be conducted immediately in the field clinic while the patient is present, the most important point for the villagers is the knowledge that they are infected with malaria parasite as well as dispersing any information for preventive measures of the disease. On the contrary, the delay in the results of microscopic diagnosis is a serious obstacles for the operation of a malaria control programme in remote areas where health staff operating the control programme have to visit several times to treat the positive subjects as per the results of microscopic examination.

Of clinical concern is the persistence of parasite antigen leading to false positives even after the clinical symptoms of malaria has disappeared and the parasites have apparently been cleared from the host as recorded earlier from central India [11,12] and world wide in a variety of settings [1]. In this study also we detected persistent positivity of RDT in 24.4% of treated patients without asexual parasitaemia on day 10. Thus the test has limited utility as a prognostic tool. Because of the relatively high rate of persistent false positive tests in recently treated cases, the value of predictability of a test band may be limited to new, untreated cases. However, the high NPV allow us to confidently diagnose negative test patients as non malaria patients in all epidemiological settings.

An additional clinical concern is the occasional failure of rapid test to detect high parasite densities as recorded earlier [10]. Pf HRP-2 deleted mutants have been reported but their prevalence is not known [13]. Unless a solution is implemented for these rare but recurrent diagnostic failure, clinicians using a Pf HRP-2 based rapid test must keep in mind that a negative test greatly reduces the probability of, but does not rule out a parasitaemia of greater than 35,000 parasites/μl. The results of more field tests may help to explain the apparent discrepancies.

In Mocha, an area free from malaria transmission, RDT may be useful to exclude the falciparum infection and indicate the need to give treatment for other causes of fever. RDT thus would improve the accuracy of the diagnosis and consequently decrease the use of antimalarials. In the medical college hospital the RDT also appeared useful although the test sensitivity was not high as in field clinics. However, RDT could facilitate the early diagnosis and an appropriate therapy in patients with cerebral malaria thereby reducing mortality as recorded earlier [7,9]. The value of the RDT specificity observed in the present study is consistent with the result of the study carried out in Uganda [5].

In addition to good sensitivity and specificity, the criteria for the rapid test should also include affordability and user friendliness. The cost per test is Rs. 28 i.e. US$ 0.50, the cheapest available so far to the Ministry of Health and the institutes buying large numbers of test kits. Besides, where the number of people to be tested is very low in small villages in remote areas, where health-facility coverage is low, and the risk of contracting malaria is high, the test may be more cost effective than microscopy as the time factor may be in favour of the rapid test.

Despite some limitations, a post study survey of all the personnel directly involved in the performance of the RDT were of opinion that the test was easy to use, decreased stress and potential delay in the diagnosis of falciparum malaria in field. If the present observations are validated in larger multicentre, clinical trial, the test may prove to be a useful alternative to microscopy, particularly in places where the facilities for microscopy are poor or non existent.

## Conclusion

RDTs in conjunction with microscopy should improve diagnosis of malaria. However, RDTs are more suited to investigators/health workers in situations where health services are deficient or absent. Therefore, it is reasonable to consider future use of the RDTs as an epidemiological tool for the rapid screening of malaria.

## Competing interests

The author(s) declare that they have no competing interests.

## Authors' contributions

Neeru Singh: Participated in design of the study and writing the manuscript.

MM Shukla: Involved in rapid analysis in Medical College Hospital, Jabalpur.

AK Mishra: Performed the rapid test in Korea district and help in analysis.

SK Chand: Involved in data collection and analysis in Mocha, Mandla.

Praveen Kumar Bharti: Performed the test in baigachak, Dindori.

All authors read and approved the final manuscript.

## Pre-publication history

The pre-publication history for this paper can be accessed here:



## References

[B1] Moody A (2002). Rapid diagnostic tests for malaria parasites. Clin Microbiol Ref.

[B2] World Health Organization (2000). Malaria diagnostics, New Perspectives. WHO/MAL.

[B3] Marsh K, English M, Peshu N, Crawley J, Snow R (1996). Clinical algorithm for malaria in Africa (letter). Lancet.

[B4] White NJ (1996). The treatment of malaria. N Engl J Med.

[B5] Guthmann JP, Ruiz A, Protto G, Kiguli J, Bonte L, Legros D (2002). Validity, reliability and ease of use in the field of five rapid tests for the diagnosis of *Plasmodium falciparum *malaria in Uganda. Trans R Soc Trop Med Hyg.

[B6] Anonymous (2003). *Annual report*. National Anti Malaria Programme Directorate of Health Services, Bhopal, Madhya Pradesh, India.

[B7] Singh N, Saxena A (2005). Usefulness of rapid on site *Plasmodium falciparum *diagnosis (Paracheck^® ^Pf) in forest migrants and among indigenous population at the site of their occupational activities in central India. Am J Trop Med Hyg.

[B8] Singh N, Valecha N (2000). Evaluation of a rapid diagnostic test 'Determine™ Malaria pf', in epidemic-prone forest villages of central India (Madhya Pradesh). Annal of Trop Med & Paraasitol.

[B9] Singh N, Nagpal AC (2004). Performance of OptiMAL dipstick test for management of severe and complicated malaria cases in a tertiary hospital, central India. J infect.

[B10] Jelinek T, Grobusch MP, Schwenke S, Steidl S, Sonnenburg FN, Nothdurft HD, Klein E, Loscher T (1999). Sensitivity and specificity of dipstick tests for rapid diagnosis of malaria in non immune travelers. J Clin Microbiol.

[B11] Singh N, Valecha N, Sharma VP (1997). Malaria diagnosis by field workers using an immunochromatographic tests. Trans R Soc Trop Med Hyg.

[B12] Singh N, Singh MP, Sharma VP (1997). The use of a dipstick antigen capture assay for the diagnosis of *Plasmodium falciparum *infection in a remote forested area of central India. Am J Trop Med Hyg.

[B13] Traore I, Koita O, Doumbo O, Kassambara L, Ouattara A, Diakite M, Sagara I, Diallo M, Krogstad DJ (1997). Field studies of the ParaSight™ F test in a malaria endemic area: cost, feasibility, sensitivity, specificity, predictive value and detection of HRP2 gene among wild type *Plasmodium falciparum *in Mali. Am J Trop Med Hyg.

